# Molecular hydrogen protects chondrocytes from oxidative stress and indirectly alters gene expressions through reducing peroxynitrite derived from nitric oxide

**DOI:** 10.1186/2045-9912-1-18

**Published:** 2011-08-04

**Authors:** Teruyasu Hanaoka, Naomi Kamimura, Takashi Yokota, Shinro Takai, Shigeo Ohta

**Affiliations:** 1 Department of Biochemistry and Cell Biology, Institute of Development and Aging Sciences, Graduate School of Medicine, Nippon Medical School, 1-396 Kosugi-machi, Nkahara-ku, Kawasaki-city, Kanagawa 211-8533, Japan; 2 Department of Orthopedic Surgery, Nippon Medical School, 1-1-5 Sendagi, Bunkyou-ku, Tokyo, 113-8602, Japan

## Abstract

**Background:**

Molecular hydrogen (H_2_) functions as an extensive protector against oxidative stress, inflammation and allergic reaction in various biological models and clinical tests; however, its essential mechanisms remain unknown. H_2 _directly reacts with the strong reactive nitrogen species peroxynitrite (ONOO^-^) as well as hydroxyl radicals (•OH), but not with nitric oxide radical (NO•). We hypothesized that one of the H_2 _functions is caused by reducing cellular ONOO^-^, which is generated by the rapid reaction of NO• with superoxides (•O_2_^-^). To verify this hypothesis, we examined whether H_2 _could restore cytotoxicity and transcriptional alterations induced by ONOO^- ^derived from NO• in chondrocytes.

**Methods:**

We treated cultured chondrocytes from porcine hindlimb cartilage or from rat meniscus fibrecartilage with a donor of NO•, *S*-nitroso-*N*-acetylpenicillamine (SNAP) in the presence or absence of H_2_. Chondrocyte viability was determined using a LIVE/DEAD Viability/Cytotoxicity Kit. Gene expressions of the matrix proteins of cartilage and the matrix metalloproteinases were analyzed by reverse transcriptase-coupled real-time PCR method.

**Results:**

SNAP treatment increased the levels of nitrated proteins. H_2 _decreased the levels of the nitrated proteins, and suppressed chondrocyte death. It is known that the matrix proteins of cartilage (including aggrecan and type II collagen) and matrix metalloproteinases (such as MMP3 and MMP13) are down- and up-regulated by ONOO^-^, respectively. H_2 _restoratively increased the gene expressions of aggrecan and type II collagen in the presence of H_2_. Conversely, the gene expressions of MMP3 and MMP13 were restoratively down-regulated with H_2_. Thus, H_2 _acted to restore transcriptional alterations induced by ONOO^-^.

**Conclusions:**

These results imply that one of the functions of H_2 _exhibits cytoprotective effects and transcriptional alterations through reducing ONOO^-^. Moreover, novel pharmacological strategies aimed at selective removal of ONOO^- ^may represent a powerful method for preventive and therapeutic use of H_2 _for joint diseases.

## Background

We have reported that molecular hydrogen (H_2_) has potential as a novel antioxidant in preventive and therapeutic applications [1]. Furthermore, H_2 _exhibits not only anti-oxidative stress effects [2,3], but also has various anti-inflammatory [4,5] and anti-allergic effects [6]. Since the publication of the first article on the biological contribution of H_2 _in 2007, more than 80 articles involved in H_2 _have been published to establish the apparent activity of H_2 _from various medical aspects [7-9].

H_2 _reacted with strong reactive oxygen/nitrogen species including hydroxyl radical and peroxinitrite (ONOO^-^) in cell-free reactions and protected cultured cells depending upon the decrease of hydroxyl radicals (**•**OH) [1]. Subsequent and recent experiments including ours indicated that a small amount of hydrogen is also effective against various stimuli [8,9]. When model animals consumed H_2 _by drinking water with dissolved H_2_, a small amount of H_2 _was extensively effective [10-12]; however, it may be difficult to explain that direct reduction of **•**OH by a very small amount of H_2 _reveals all the functions of H_2_, because the saturated level of H_2 _is only 0.8 mM and the dwelling time of **•**OH is very short in the body [11,13]. In fact, drinking 0.04 or 0.08 mM H_2 _was shown to be effective [14,15]. Although we have recently shown that H_2 _can be accumulated with hepatic glycogen, it is unlikely that the amount of H_2 _is sufficient to exhibit all of its functions [15].

Moreover, H_2 _regulated various gene expressions; however, there is no evidence that H_2 _directly reacts with factors involved in transcriptional regulation including FGF21 [15], inflammatory cytokines [11], HMGB1 [16], and HO-1 [17]. It remains unclear whether such regulations are the cause or consequence of the effects against oxidative stress. Moreover, the primary molecular target of H_2 _remains unknown.

ONOO^- ^is produced by the rapid reaction of nitric monoxide (NO**•) **with superoxide anion radicals (**•**O_2_^-^) [18,19]. We have shown that H_2 _reduces ONOO^- ^as well as **•**OH [1]. Different from **•**OH, ONOO^- ^has a longer lifespan and the potential to regulate gene expression through nitration of target proteins [20,21]. Thus, we hypothesized that one of the H_2 _functions is caused by reducing cellular ONOO^-^.

Here, to verify this hypothesis, we examined protective and regulatory effects of H_2 _on NO•-derived oxidative stress to chondrocytes. We found that H_2 _protected chondrocytes from oxidative stress, and alternated gene expressions, contrary to the manner of transcriptional regulation by ONOO^-^. This study implies that at least one of the H_2 _functions is responsible for the reduction of ONOO^-^.

## Methods

### Cartilage slice culture

A fresh hindlimb of a slaughtered male seven-month-old pig was purchased from Tokyo Shibaura Organ Co., Ltd. (Minato-ku, Tokyo, Japan). There were no possible contaminant diseases. Cartilage from the healthy porcine hindlimb (metatarsophalangeal joint) was cut into pieces for culture (2 mm width × 7 mm length × full thickness) as described previously [22]. Male Sprague-Dawley rats of 10 weeks of age were purchased from Nippon SLC (Hamamatsu, Shizuoka, Japan). Cartilage from the meniscus of a rat was also sliced into pieces (full width × full length × 0.5 mm thickness) for culture. Since the meniscus structure is not uniform and the peripheral part contains fewer chondrocytes, we used slices prepared from the middle part of the meniscus.

The slices were randomly divided into two experimental groups and incubated at 37°C in Dulbecco's modified Eagle's medium (DMEM)/Ham F-12 mixed medium (Gibco Invitrogen, Grand Island, NY, USA) supplemented with 10% fetal calf serum (FBS), penicillin (100 U/ml), and streptomycin (100 μg/ml).

The care and use of laboratory animals were in accordance with the NIH guidelines. This study was approved by the Animal Care and Use Committee of Nippon Medical School (Bunkyo-ku, Tokyo, Japan).

### Hydrogen treatment

We prepared H_2_-dissolved culture medium as described previously [1]. In brief, we dissolved H_2 _in the medium by bubbling H_2 _gas to the saturated level. We also dissolved O_2 _in a second medium by bubbling O_2 _gas, and CO_2 _in a third medium by bubbling CO_2 _gas. We combined these media to give a medium consisting of 75% H_2_, 20% O_2_, 5% CO_2 _(vol/vol/vol). We then cultured the cartilage slices in a closed culture flask filled with the medium. Control medium contained 75% N_2 _instead of H_2_. The H_2 _concentration was maintained for 24 hr as described [15].

### Cell death assay

The cartilage slices were incubated for 12 - 80 hr in medium containing 0.3 - 3 mM *S*-nitroso-*N*-acetyl-D, L-penicillamine (SNAP) (Cayman Chemical, Ann Arbor, MI, USA) in the presence or absence of H_2 _[22,23]. Chondrocyte viability was determined using a LIVE/DEAD Viability/Cytotoxicity Kit (Molecular Probes, Eugene, OR, USA). Living, dying and dead cells were stained with green, yellow (combination of green and red) and red fluorescence, respectively, and visualized with a confocal scanning laser microscope (FLUOVIEW FV300; Olympus, Tokyo, Japan).

### Immunohistochemical staining

Frozen sections of 6 μm-thick were fixed with 10% formalin and treated with 0.3% hydrogen peroxide in methanol to inhibit endogenous peroxidase activity. The sections were incubated with 10% Block Ace (DS Pharma Biomedical Co., Ltd., Suita, Osaka, Japan) in phosphate buffered saline (PBS) and then incubated with anti-nitrotyrosine monoclonal antibody (Calbiochem, San Diego, CA, USA; 1:100 dilution with 10% Block Ace in PBS) overnight at 4°C. Nitrotyrosine residues were visualized with DAB using horseradish peroxidase (HRP)-conjugated secondary antibody (Santa Cruz Biotechnology, Inc. Santa Cruz, CA, USA) and a HistoMark ORANGE kit (KPL, Gaithersburg, MD, USA). As a positive control for staining, we used sections from cartilage treated with 1 mM 3-morpholinosydnonimine (SIN-1) (Sigma-Aldrich, St. Louis, MO, USA), which generates both superoxide anion and nitric oxide that spontaneously produce peroxynitrite. The positive area was estimated using the Image J program (version 1.41; National Institutes of Health, Bethesda, MD, USA) from four sections for each group.

### RNA isolation and RT-PCR

Total RNA was isolated from the cartilage using an RNeasy Mini kit (QIAGEN, Valencia, CA, USA). Complementary DNA synthesized by SuperScript II Reverse Transcriptase (Invitrogen, Carlsbad, CA, USA) was analyzed by quantitative PCR using the Thermal Cycler Dice Real Time System TP800 (TAKARA BIO Inc., Otsu, Shiga, Japan). All samples were normalized to glyceraldehyde 3-phosphate dehydrogenase (GAPDH) expression. Primer and probe sequences for each PCR are listed in Table 1.

**Table 1:** Primers and probes for RT-PCR.

Gene		Sequence
aggrecan	F primer	5'-GACCAGGAGCAATGTGAGGAG-3'
	R primer	5'-CTCGCGGTCGGGAAAGT-3'
	probe	5'-CCAAGTTCCAGGGCCACTGTTATCGC-3'
type II collagen	F primer	5'-TTGGAGAGACCATGAACGGC-3'
	R primer	5'-TTAGCGGTGTTGGGAGCC-3'
	probe	5'-CACTTCAGCTACGGCGACGGCAA-3'
MMP3	F primer	5'-TCCCAGGAAAATAGCTGAGAACTT-3'
	R primer	5'-AAACCCAAATGCTTCAAAGACAG-3'
	probe	5'-CCAGGCATTGGCACAAAGGTGGA-3'
MMP13	F primer	5'-TGGAGTTATGATGATGCTAACCAGAC-3'
	R primer	5'-TGTCGCCAATTCCAGGGA-3'
	probe	5'-TGGACAAAGACTATCCCCGCCTCATAGAAG-3'
GAPDH	F primer	5'-CATCACTGCCACCCAGAAGA-3'
	R primer	5'-ATGTTCTGGGCAGCC-3'
	probe	5'-TGGATGGCCCCTCTGGAAAGCTG-3'

### Immunoblot analysis

Specimens were homogenized with a micro-homogenizer in SDS (sodium dodecyl sulfate) buffer (1% SDS in PBS), and then centrifuged at 10,000 g for 10 min at 4°C to remove debris. Supernatants were subjected to SDS-PAGE (SDS-polyacrylamide gel electrophoresis) followed by electrotransfer onto a PVDF membrane. The blotted membranes were blocked with Block Ace (DS Pharma Biomedical Co., Ltd.) and incubated with anti-aggrecan polyclonal antibody (Abcam, Cambridge, UK; 1:1,000 dilution), anti-MMP13 polyclonal antibody (Santa Cruz Biotechnology, Inc. 1:1,000 dilution) or anti-actin monoclonal antibody (Sigma-Aldrich; 1:500 dilution) overnight at 4°C. Each band was visualized with horseradish peroxidase (HRP)-conjugated secondary antibody (Santa Cruz Biotechnology, Inc.) and an ECL plus Western blotting detection system (GE Healthcare, Piscataway, NJ, USA).

### Statistical analysis

We performed statistical analysis using StatView software (SAS Institute) by applying an unpaired two-tailed Student's t-test and ANOVA followed by Fisher's exact test, as described previously [1]. Differences were considered significant at p < 0.05.

## Results

### H_2 _protects chondrocytes of hyaline and fibrecartilage from cell death

**Figure 1:**
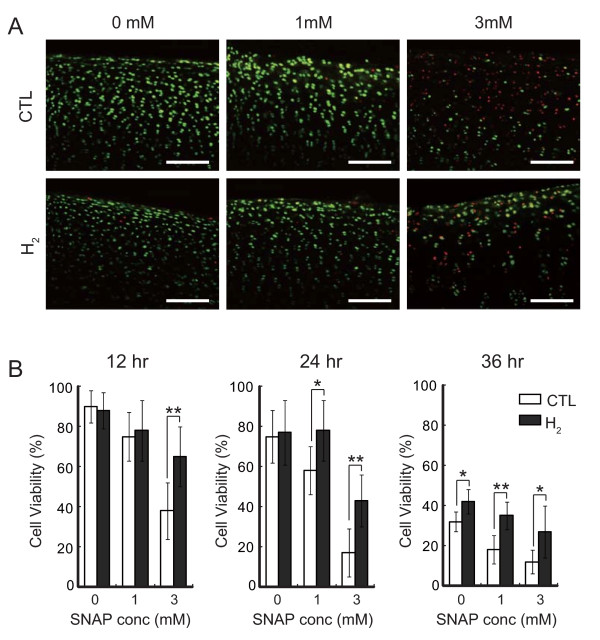
**Hydrogen protects chondrocytes of hyaline cartilage from cell death**. (A) Porcine cartilage slices were incubated with 0, 1 or 3 mM SNAP in the presence or absence of hydrogen for 12 hr at 37°C. Cells were stained with a mixture of calcein AM (Live cell: green) and ethidium homodimer (Dead cell: red) as described in Materials and methods. Scale bar: 100 μm. (B) Chondrocyte viability was determined by counting green and red cells from three areas of each slice. Six slices were used for each experimental group. The slices were incubated with 0, 1 or 3 mM SNAP in the presence or absence of hydrogen for 12, 24 or 36 hr at 37°C. Data are the means ± SD (*n *= 6). *p < 0.05; **p < 0.01.

It is reported that cultured chondrocytes are sensitive to exposure to SNAP, a donor of NO• [22] and that H_2 _exhibits no direct reaction with NO• in cultured cells as well as in a cell-free reaction. To verify the hypothesis that H_2 _protects cells by reducing ONOO^-^, we examined the effect of H_2 _on cell death induced by SNAP by using hyaline cartilage slices from a porcine hindlimb metatarsophalangeal joint as a target. Chondrocyte viability was determined using a LIVE/DEAD Viability/Cytotoxicity Kit, which provides quantitative analyses of the proportion of live and dead cells in a mixed population (Figure 1A). In living cells, membrane-permeated calcein AM is cleaved by esterases to yield cytoplasmic green fluorescence, and in dead cells membrane-impermeable ethidium homodimer-1 labels nucleic acids with red fluorescence. Dying cells, whose membrane structure has been disrupted but still have some esterase activity, were double stained as yellow. We counted green, red and yellow cells for statistical analysis (Additional file 1: Table S1). Cell viability was calculated as the percentage of green cell numbers against total cell numbers (Figure 1B). Significant protection of chondrocytes by H_2 _was observed in the treatment with 3 mM SNAP for 12 hr (Figure 1A and 1B). More evident effects were obtained with longer SNAP treatment (Figure 1B).

**Figure 2:**
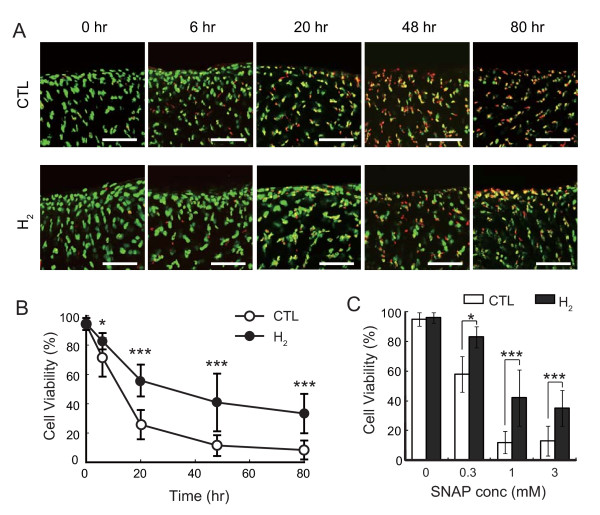
**Hydrogen protects chondrocytes of fibrocartilages from cell death**. (A) Meniscus fibrocartilages from SD rats was incubated with 1 mM SNAP in the presence or absence of hydrogen for 0, 6, 20, 48, or 80 hr at 37°C. Cells were stained with calcein AM (Live cell: green) and ethidium homodimer (Dead cell: red) as described in Materials and methods. Scale bar: 40 μm. (B) Chondrocyte viability was determined by counting green and red cells from three regions of each slice. Six slices were used for each experimental group. The slices were incubated with 1 mM SNAP in the presence or absence of hydrogen for the indicated periods at 37°C. Data are the means ± SD (*n *= 6). *p < 0.05; ***p < 0.001. (C) The slices were incubated with 0, 0.3, 1, or 3 mM SNAP in the presence or absence of hydrogen for 48 hr at 37°C. Data are the means ± SD (*n *= 6). *p < 0.05; ***p < 0.001.

Next, we examined another type of cartilage, meniscus fibrecartilage, isolated from rats instead of swine specimens. Because it is easier to isolate swine than rat cartilage, we used swine cartilage for preliminary experiments; however, for further analysis, cartilage from rats is more suitable for RNA and protein analysis because genomic databases and antibodies are available. Treatment with 1 mM SNAP induced cell death in a time-dependent manner and H_2 _suppressed chondrocyte death at each time point (Figure 2A and 2B, Additional file 2: Table S2). H_2 _significantly protected chondrocytes from death with various concentrations of SNAP treatment for 48 hr (Figure 2C, Additional file 3: Table S3). These results indicate that H_2 _protects chondrocytes by stimuli derived from NO•, although H_2 _has no potential to react with NO•.

### H_2 _decreases nitrotyrosine in chondrocytes and matrix of cartilage induced by SNAP

**Figure 3:**
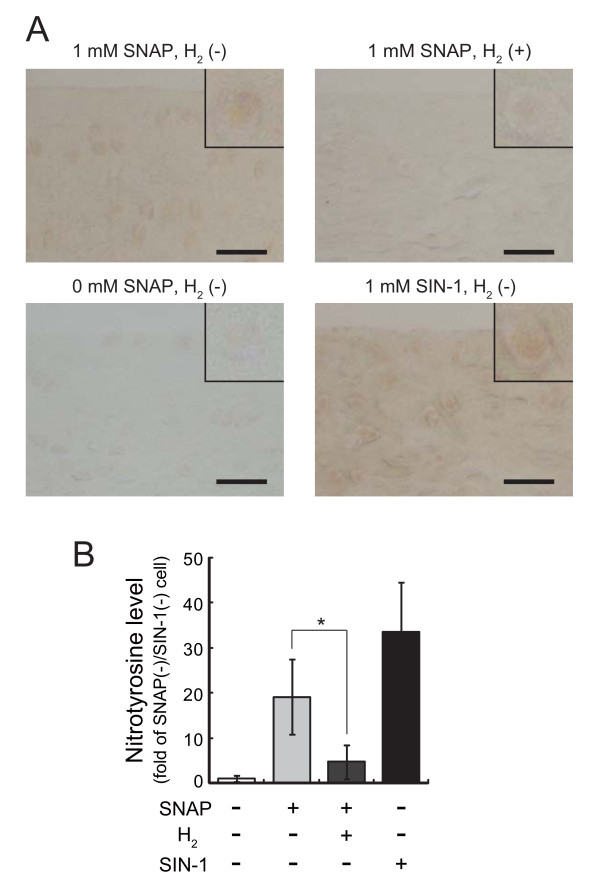
**Hydrogen decreases nitrotyrosine in chondrocytes and cartilage matrix**. (A) Meniscus fibrocartilage from SD rats was incubated with 1 mM SNAP in the presence or absence of hydrogen for 3 hr at 37°C. Frozen sections were stained with anti-nitorotyrosine antibody and visualized with DAB as described in Methods. The inset shows chondrocytes. Cartilage incubated with 1 mM SIN-1 and without SNAP was used for positive and negative staining controls, respectively. Scale bar: 40 μm. (B) Levels of nitrotyrosine in cartilage were estimated from anti-nitrotyrosine immunostaining using an image analysis program, Image J program. Data are the mean ± SD (n = 4). *p < 0.05; control versus hydrogen in 1 mM SNAP-treated groups.

ONOO^- ^is a strong modifier of nitration in proteins. To confirm that H_2 _decreased ONOO^- ^derived from NO•, we examined levels of nitrotyrosine residues in cartilage immunohistologically. In fact, NO• increased the levels of nitrotyrosine, and H_2 _restored its increase (Figure 3). Thus, H_2 _should decrease ONOO^- ^derived from NO•.

### H_2 _restores down-regulation of matrix expression and up-regulation of matrix-metallo protease expression induced by SNAP

**Figure 4:**
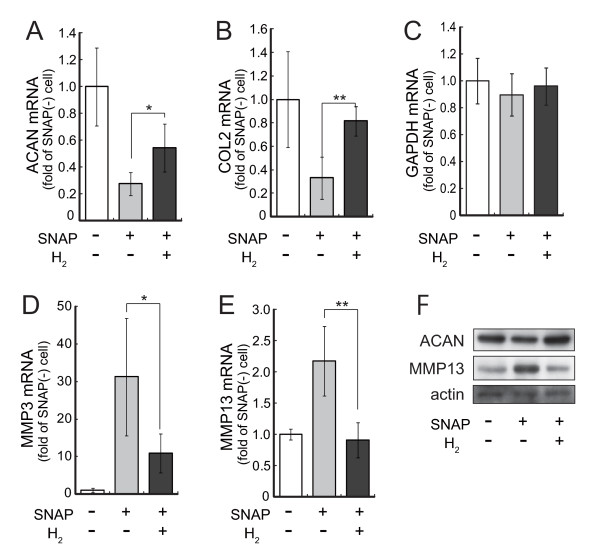
**Hydrogen alters mRNA and protein expressions of matrix proteins and matrix-metalloproteases (MMPs)**. Meniscus fibrocartilage from rats was incubated with 1 mM SNAP in the presence or absence of hydrogen for 4 hr or 20 hr at 37°C. Total RNA was extracted from 4 hr-incubated cartilage and the expression levels of aggrecan (A), type II collagen (B), GAPDH (C), MMP3 (D) and MMP13 (E) were analyzed by real-time PCR coupled with reverse transcription. Data are the mean ± SD (n = 4). *p < 0.05; **p < 0.01. (F) Total protein was extracted from 20 hr-incubated cartilage and the expression levels of aggrecan, MMP13 and actin were analyzed by immunoblotting.

It was also reported that ONOO^- ^down-regulates gene expressions of the cartilage matrix proteins including aggrecan and type II collagen [24]. Conversely, levels of matrix-metallo protease are known to be up-regulated by ONOO^- ^[24]. We then investigated the effect of H_2 _on the expression of chondrocyte-specific matrix genes. Isolated meniscus fibrecartilage was incubated in DMEM/F-12 supplemented with 10% FBS with or without 1 mM SNAP in the presence or absence of H_2_. The levels of mRNA for the matrix proteins of type II collagen and aggrecan core protein were quantified with real-time PCR coupled with reverse transcription (Figure 4A and 4B). Indeed, SNAP down-regulated aggrecan and collagen II gene expressions as expected. The decreased gene expressions of the matrix proteins were significantly restored by H_2_-dissolved culture medium, suggesting that the decreased ONOO^- ^restored the gene expression.

The possibility cannot be ruled out that oxidative damage derived from SNAP may reduce any gene expressions in a non-specific manner. We examined therefore the effect of H_2 _on catabolic enzyme genes induced by SNAP, because levels of matrix-metallo protease are known to be contradictorily up-regulated by ONOO^- ^[24]. The levels of MMP-3 and MMP-13 mRNA were measured with quantitative real-time PCR after treatment with SNAP with or without H_2 _(Figure 4D and 4E). Moreover, the alterations of the aggrecan and MMP-13 proteins corresponded to their mRNA levels (Figure 4F). Thus, SNAP up-regulated MMP-3 and MMP-13 gene expressions as expected, whereas H_2 _significantly suppressed MMP gene expressions, suggesting that H_2 _restored the increased expressions by decreasing ONOO^-^.

## Discussion

Joint diseases including osteoarthritis (OA) and rheumatoid arthritis (RA) are the most common disabling diseases, especially among elderly people. Arthritis is a degenerative disease involving abnormalities in chondrocytes, articular cartilage and other joint tissue, and is mediated by a number of underlying biochemical and physical stimuli [25,26]. Recent studies revealed that oxidative stress plays a leading role in the initiation and progression of the disease process [27,28]. As a joint disease model of aged patients, we stimulated chondrocytes with oxidative stress derived from NO•. The cartilage consists mostly of the extracellular matrix, which is synthesized by chondrocytes [28,29]. The extracellular matrix is composed of collagens and proteoglycans that are responsible for the important compressive and tensile properties of cartilage [28].

The major oxidative stress generated by chondrocytes is one of the most powerful oxidants ONOO^-^, which was produced by the rapid reaction of NO• with •O_2_^- ^[18,19]. At an earlier stage, NO• has been considered as the primary inducer of chondrocyte death [30]; however, it has been revealed that the oxidative strength of NO• is not sufficient to initiate cell death [31,32]. A series of experiments have indicated that the major cytotoxicity attributed to NO• is rather due to ONOO^- ^[20,33]. Increased ONOO^- ^formation has been observed in cartilage and subchondral bone in rodent models [34-36] and in cartilage in OA and RA patients [37-39]. ONOO^- ^induces cell death and regulates the decreased expression of collagens and proteoglycans and increased matrix metallo proteinases in chondrocytes, resulting in matrix degradation [24,40]. Thus, chondrocyte is a suitable target for investing the effect of H_2 _regarding ONOO^- ^in this study.

In this study, we show that H_2 _protected chondrocytes from death induced by SNAP. SNAP is a donor of NO•; however, NO• has no strong toxicity itself and H_2 _has no potential to reduce NO•. Our previous study demonstrated that H_2 _reduces ONOO^- ^in a cell-free system [1]. Thus, we speculate that H_2 _would protect SNAP-treated chondrocytes by decreasing ONOO^-^. More importantly, it has been reported that drinking hydrogen water suppress the nitration of kidney proteins, although H_2 _received from hydrogen water remained for only short period in the organ (less than 5 min) [11]. In this study, we have shown that H_2 _in medium suppress the nitration of the chondrocyte proteins (Figure 3). Thus, it is possible that even a very small amount of H_2 _exhibits anti-oxidative effects by reducing ONOO^- ^in many situations.

Several laboratories including ours have reported that H_2 _altered gene expressions involved in inflammation or energy metabolism when animals drank hydrogen water [15,17]; however, it is an open question why H_2 _alters gene expressions, because there is no evidence that H_2 _directly influences gene expressions. On the other hand, ONOO^- ^has the potential to regulate gene expressions through the nitration of factors involved in transcriptional regulation [20]. As mentioned above, drinking hydrogen water suppresses the nitration of proteins; thus, it is possible that the very small amount of H_2 _consumed by drinking hydrogen water influences nitration in *in vivo *experiments and results in regulatory as well as anti-oxidative effects [11]. These results agree with the present finding that H_2 _suppressed the nitration of proteins.

Taken together, this study implies that one of the H_2 _functions, including transcriptional alterations, is caused through reducing ONOO^- ^derived from NO•.

Novel pharmacological strategies aimed at selective removal of ONOO^- ^may represent a powerful method for preventive and therapeutic use of H_2 _for joint diseases. Cartilage has no blood vessels and nutrients are supplied through fluid. Since H_2 _has a great advantage to rapidly diffuse into tissues even without blood flow [41,42], it may be useful to prevent joint diseases by reducing oxidative stress and by suppressing the decrease in matrix proteins and inhibiting degradation by proteinases.

## Conclusions

This study implies that one of the H_2 _functions, including transcriptional alterations, is caused through reducing ONOO^- ^derived from NO•. Novel pharmacological strategies aimed at selective removal of ONOO^- ^may represent a powerful method for preventive and therapeutic use of H_2 _for joint diseases.

## List of abbreviations

SNAP: *S*-nitroso-*N*-acetylpenicillamine; MMP: matrix metalloproteinase; GAPDH: glyceraldehyde 3-phosphate dehydrogenase; OA: osteoarthritis; RA: rheumatoid arthritis.

## Competing interests

The authors declare that they have no competing interests.

## Authors' contributions

SO and ST conceived the experiments. SO and NK designed the actual experiments. TH, TY and NK performed the experiments and data analysis. NK and SO interpreted the data and wrote the paper. All authors have been involved in drafting the manuscript it critically for important intellectual content; and have given final approval of the version to be published.

## Supplementary Material

Additional file 1 **Table S1 - Live, dying, and dead cell numbers of hyaline cartilage**. Click here for file

Additional file 2 **Table S2 - Live, dying, and dead cell numbers of fibrocartilages treated with 1 mM SNAP**. Click here for file

Additional file 3 **Table S3 - Live, dying, and dead cell numbers of fibrocartilages treated with various concentration of SNAP for 48 hr**. Click here for file

## References

[B1] Ohsawa I Ishikawa M Takahashi K Watanabe M Nishimaki K Yamagata K Katsura K Katayama Y Asoh S Ohta S Hydrogen acts as a therapeutic antioxidant by selectively reducing cytotoxic oxygen radicals Nat Med 2007 13 688 694 10.1038/nm1577 17486089

[B2] Fukuda K Asoh S Ishikawa M Yamamoto Y Ohsawa I Ohta S Inhalation of hydrogen gas suppresses hepatic injury caused by ischemia/reperfusion through reducing oxidative stress Biochem Biophys Res Commun 2007 361 670 674 10.1016/j.bbrc.2007.07.088 17673169

[B3] Ohsawa I Nishimaki K Yamagata K Ishikawa M Ohta S Consumption of hydrogen water prevents atherosclerosis in apolipoprotein E knockout mice Biochem Biophys Res Commun 2008 377 1195 1198 10.1016/j.bbrc.2008.10.156 18996093

[B4] Kajiya M Silva MJ Sato K Ouhara K Kawai T Hydrogen mediates suppression of colon inflammation induced by dextran sodium sulfate Biochem Biophys Res Commun 2009 386 11 15 10.1016/j.bbrc.2009.05.117 19486890

[B5] Kajiya M Sato K Silva MJ Ouhara K Do PM Shanmugam KT Kawai T Hydrogen from intestinal bacteria is protective for Concanavalin A-induced hepatitis Biochem Biophys Res Commun 2009 386 316 321 10.1016/j.bbrc.2009.06.024 19523450

[B6] Itoh T Fujita Y Ito M Masuda A Ohno K Ichihara M Kojima T Nozawa Y Molecular hydrogen suppresses FcepsilonRI-mediated signal transduction and prevents degranulation of mast cells Biochem Biophys Res Commun 2009 389 651 656 10.1016/j.bbrc.2009.09.047 19766097

[B7] Huang CS Kawamura T Toyoda Y Nakao A Recent advances in hydrogen research as a therapeutic medical gas Free Radic Res 2010 44 971 982 10.3109/10715762.2010.500328 20815764

[B8] Ohta S Molecular hydrogen is a novel antioxidant to efficiently reduce oxidative stress with potential for the improvement of mitochondrial diseases Biochim Biophys Acta 2011 in press 10.1016/j.bbagen.2011.05.006 21621588

[B9] Ohta S Recent progress toward hydrogen medicine: Potential of molecular hydrogen for preventive and therapeutic applications Curr Pharm Des 2011 in press 10.2174/138161211797052664 PMC3257754 21736547

[B10] Nagata K Nakashima-Kamimura N Mikami T Ohsawa I Ohta S Consumption of molecular hydrogen prevents the stress-induced impairments in hippocampus-dependent learning tasks during chronic physical restraint in mice Neuropsychopharmacology 2009 34 501 508 10.1038/npp.2008.95 18563058

[B11] Cardinal JS Zhan J Wang Y Sugimoto R Tsung A McCurry KR Billiar TR Nakao A Oral hydrogen water prevents chronic allograft nephropathy in rats Kidney Int 2010 77 101 109 10.1038/ki.2009.421 19907413

[B12] Fu Y Ito M Fujita Y Ichihara M Masuda A Suzuki Y Maesawa S Kajita Y Hirayama M Ohsawa I Ohta S Ohno K Molecular hydrogen is protective against 6-hydroxydopamine-induced nigrostriatal degeneration in a rat model of Parkinson's disease Neurosci Lett 2009 453 81 85 10.1016/j.neulet.2009.02.016 19356598

[B13] Nakashima-Kamimura N Mori T Ohsawa I Asoh S Ohta S Molecular hydrogen alleviates nephrotoxicity induced by an anti-cancer drug cisplatin without compromising anti-tumor activity in mice Cancer Chemother Pharmacol 2009 64 753 761 10.1007/s00280-008-0924-2 19148645

[B14] Fujita K Seike T Yutsudo N Ohno M Yamada H Yamaguchi H Sakumi K Yamakawa Y Kido MA Takaki A Katafuchi T Tanaka Y Nakabeppu Y Noda M Hydrogen in drinking water reduces dopaminergic neuronal loss in the 1-methyl-4-phenyl-1,2,3,6-tetrahydropyridine mouse model of Parkinson's disease PLoS One 2009 4 e7247 10.1371/journal.pone.0007247 19789628 PMC2747267

[B15] Kamimura N Nishimaki K Ohsawa I Ohta S Molecular hydrogen improves obesity and diabetes by inducing hepatic FGF21 and stimulating energy metabolism in db/db mice Obesity 2011 in press 10.1038/oby.2011.6 21293445

[B16] Xie K Yu Y Pei Y Hou L Chen S Xiong L Wang G Protective effects of hydrogen gas on murine polymicrobial sepsis via reducing oxidative stress and HMGB1 release Shock 2010 34 90 97 19997046 10.1097/SHK.0b013e3181cdc4ae

[B17] Huang CS Kawamura T Lee S Tochigi N Shigemura N Buchholz BM Kloke JD Billiar TR Toyoda Y Nakao A Hydrogen inhalation ameliorates ventilator-induced lung injury Crit Care 2010 14 R234 10.1186/cc9389 21184683 PMC3219999

[B18] Huie RE Padmaja S The reaction of no with superoxide Free Radic Res Commun 1993 18 195 199 10.3109/10715769309145868 8396550

[B19] Beckman JS Beckman TW Chen J Marshall PA Freeman BA Apparent hydroxyl radical production by peroxynitrite: implications for endothelial injury from nitric oxide and superoxide Proc Natl Acad Sci USA 1990 87 1620 1624 10.1073/pnas.87.4.1620 2154753 PMC53527

[B20] Pacher P Beckman JS Liaudet L Nitric oxide and peroxynitrite in health and disease Physiol Rev 2007 87 315 424 10.1152/physrev.00029.2006 17237348 PMC2248324

[B21] Klotz LO Schroeder P Sies H Peroxynitrite signaling: receptor tyrosine kinases and activation of stress-responsive pathways Free Radic Biol Med 2002 33 737 743 10.1016/S0891-5849(02)00892-4 12208362

[B22] Ozaki D Sudo K Asoh S Yamagata K Ito H Ohta S Transduction of anti-apoptotic proteins into chondrocytes in cartilage slice culture Biochem Biophys Res Commun 2004 313 522 527 10.1016/j.bbrc.2003.11.144 14697220

[B23] Sudo K Asoh S Ohsawa I Ozaki D Yamagata K Ito H Ohta S The anti-cell death FNK protein protects cells from death induced by freezing and thawing Biochem Biophys Res Commun 2005 330 850 856 10.1016/j.bbrc.2005.03.059 15809074

[B24] Henrotin YE Bruckner P Pujol JP The role of reactive oxygen species in homeostasis and degradation of cartilage Osteoarthritis Cartilage 2003 11 747 755 10.1016/S1063-4584(03)00150-X 13129694

[B25] Roach HI Aigner T Soder S Haag J Welkerling H Pathobiology of osteoarthritis: pathomechanisms and potential therapeutic targets Curr Drug Targets 2007 8 271 282 10.2174/138945007779940160 17305505

[B26] Ge Z Hu Y Heng BC Yang Z Ouyang H Lee EH Cao T Osteoarthritis and therapy Arthritis Rheum 2006 55 493 500 10.1002/art.21994 16739189

[B27] Halliwell B Oxygen radicals, nitric oxide and human inflammatory joint disease Ann Rheum Dis 1995 54 505 510 10.1136/ard.54.6.505 7632097 PMC1009913

[B28] Henrotin Y Kurz B Aigner T Oxygen and reactive oxygen species in cartilage degradation: friends or foes? Osteoarthritis Cartilage 2005 13 643 654 10.1016/j.joca.2005.04.002 15936958

[B29] Muir H The chondrocyte, architect of cartilage. Biomechanics, structure, function and molecular biology of cartilage matrix macromolecules Bioessays 1995 17 1039 1048 10.1002/bies.950171208 8634065

[B30] Blanco FJ Ochs RL Schwarz H Lotz M Chondrocyte apoptosis induced by nitric oxide Am J Pathol 1995 146 75 85 7856740 PMC1870754

[B31] Del Carlo M Loeser RF Nitric oxide-mediated chondrocyte cell death requires the generation of additional reactive oxygen species Arthritis Rheum 2002 46 394 403 10.1002/art.10056 11840442

[B32] Clements KM Burton-Wurster N Lust G The spread of cell death from impact damaged cartilage: lack of evidence for the role of nitric oxide and caspases Osteoarthritis Cartilage 2004 12 577 585 10.1016/j.joca.2004.04.006 15219573

[B33] Whiteman M Armstrong JS Cheung NS Siau JL Rose P Schantz JT Jones DP Halliwell B Peroxynitrite mediates calcium-dependent mitochondrial dysfunction and cell death via activation of calpains FASEB J 2004 18 1395 1397 15240564 10.1096/fj.03-1096fje

[B34] Cuzzocrea S Chatterjee PK Mazzon E McDonald MC Dugo L Di Paola R Serraino I Britti D Caputi AP Thiemermann C Beneficial effects of GW274150, a novel, potent and selective inhibitor of iNOS activity, in a rodent model of collagen-induced arthritis Eur J Pharmacol 2002 453 119 129 10.1016/S0014-2999(02)02338-5 12393067

[B35] Szabo C Virag L Cuzzocrea S Scott GS Hake P O'Connor MP Zingarelli B Salzman A Kun E Protection against peroxynitrite-induced fibroblast injury and arthritis development by inhibition of poly(ADP-ribose) synthase Proc Natl Acad Sci USA 1998 95 3867 3872 10.1073/pnas.95.7.3867 9520459 PMC19929

[B36] Yonekura Y Koshiishi I Yamada K Mori A Uchida S Nakamura T Utsumi H Association between the expression of inducible nitric oxide synthase by chondrocytes and its nitric oxide-generating activity in adjuvant arthritis in rats Nitric Oxide 2003 8 164 169 10.1016/S1089-8603(03)00025-9 12826065

[B37] Loeser RF Carlson CS Del Carlo M Cole A Detection of nitrotyrosine in aging and osteoarthritic cartilage: Correlation of oxidative damage with the presence of interleukin-1beta and with chondrocyte resistance to insulin-like growth factor 1 Arthritis Rheum 2002 46 2349 2357 10.1002/art.10496 12355482

[B38] Kaur H Halliwell B Evidence for nitric oxide-mediated oxidative damage in chronic inflammation. Nitrotyrosine in serum and synovial fluid from rheumatoid patients FEBS Lett 1994 350 9 12 10.1016/0014-5793(94)00722-5 8062931

[B39] Sandhu JK Robertson S Birnboim HC Goldstein R Distribution of protein nitrotyrosine in synovial tissues of patients with rheumatoid arthritis and osteoarthritis J Rheumatol 2003 30 1173 1181 12784386

[B40] Abramson SB Osteoarthritis and nitric oxide Osteoarthritis Cartilage 2008 16 Suppl 2 S15 20 18794013 10.1016/S1063-4584(08)60008-4

[B41] Hayashida K Sano M Ohsawa I Shinmura K Tamaki K Kimura K Endo J Katayama T Kawamura A Kohsaka S Makino S Ohta S Ogawa S Fukuda K Inhalation of hydrogen gas reduces infarct size in the rat model of myocardial ischemia-reperfusion injury Biochem Biophys Res Commun 2008 373 30 35 10.1016/j.bbrc.2008.05.165 18541148

[B42] Oharazawa H Igarashi T Yokota T Fujii H Suzuki H Machide M Takahashi H Ohta S Ohsawa I Protection of the retina by rapid diffusion of hydrogen: administration of hydrogen-loaded eye drops in retinal ischemia-reperfusion injury Invest Ophthalmol Vis Sci 2010 51 487 492 10.1167/iovs.09-4089 19834032

