# Supplementary material for: Molecular hydrogen protects chondrocytes from oxidative stress and indirectly alters gene expressions through reducing peroxynitrite derived from nitric oxide
Source: Med Gas Res. 2011 Aug 4;1:18. doi: 10.1186/2045-9912-1-18 (PMC3231990; doi:10.1186/2045-9912-1-18)
Supplement: Additional file 3 — Table S3 - Live, dying, and dead cell numbers of fibrocartilages treated with various concentration of SNAP for 48 hr. [file 2045-9912-1-18-S3.PDF]

Table S3. Live, dying, and dead cell numbers of fibrocartilages treated with various concentration of SNAP for 48 hr.

SNAP conc	CTL			H_2		
(mM)	live	dying	dead	live	dying	dead
0	37.2±1.7	1.2±0.4	0.9±0.3	33.9±1.2**	1.1±0.2	0.9±0.3
0.3	22.3±4.7	10.8±2.1	5.4±1.6	29.5±2.4**	3.7±0.9***	2.8±0.6**
1.0	4.7±2.9	12.1±4.5	21.7±9.0	14.7±6.6**	7.3±6.7	13.2±9.7
3.0	5.1±3.9	5.4±2.7	29.4±9.4	12.2±4.2*	4.5±3.2	18.3±7.8

Cartilage were stained with LIVE/DEAD kit as described in Materials and Methods section and the numbers of green (live), yellow (double stained dying cell), and red (dead) cells were counted from three areas (6400 μm^2) of each slice. Six slices were used for each experimental group. The slices were incubated with 0, 0.3, 1.0 or 3.0 mM SNAP in the presence or absence of hydrogen for 48 hr at 37 °C. Data are the means ± SD ($n = 6$). *$p < 0.05$; **$p < 0.01$; ***$p < 0.001$; control versus hydrogen.
